# Use and mis-use of supplementary material in science publications

**DOI:** 10.1186/s12859-015-0668-z

**Published:** 2015-11-03

**Authors:** Mihai Pop, Steven L. Salzberg

**Affiliations:** Department of Computer Science and Center for Bioinformatics and Computational Biology, University of Maryland, College Park, MD 20742 USA; Center for Computational Biology McKusick-Nathans Institute of Genetic Medicine, Johns Hopkins University School of Medicine, Baltimore, MD 21205 USA

## Abstract

Supplementary material is a ubiquitous feature of scientific articles, particularly in journals that limit the length of the articles. While the judicious use of supplementary material can improve the readability of scientific articles, its excessive use threatens the scientific review process and by extension the integrity of the scientific literature. In many cases supplementary material today is so extensive that it is reviewed superficially or not at all. Furthermore, citations buried within supplementary files rob other scientists of recognition of their contribution to the scientific record. These issues are exacerbated by the lack of guidance on the use of supplementary information from the journals to authors and reviewers. We propose that the removal of artificial length restrictions plus the use of interactive features made possible by modern electronic media can help to alleviate these problems. Many journals, in fact, have already removed article length limitations (as is the case for BMC Bioinformatics and other BioMed Central journals). We hope that the issues raised in our article will encourage publishers and scientists to work together towards a better use of supplementary information in scientific publishing.

**Electronic supplementary material:**

The online version of this article (doi:10.1186/s12859-015-0668-z) contains supplementary material, which is available to authorized users.

## Introduction

Supplementary material is ubiquitous in scientific papers. For example, in the most recent issues of *Science* and *Nature*, every single paper contains supplementary information (data and/or text) that does not appear in the print version of the journal. Primarily used to circumvent page limits imposed by journals, supplementary material can in some instances help improve the presentation, even in papers not subjected to length limitations. For example, a manuscript might present a high-level view of the methods employed in the analysis while detailed technical descriptions of the methods (essential for ensuring reproducibility) can be relegated to an online supplement. As a result, the story presented in the main manuscript can be laid out in a more concise and clear fashion, while still allowing interested readers to drill down into the details of the analysis. When used appropriately, supplementary material made available as an online companion to a paper provides scientific authors and publishers the means to achieve a compromise between readability and reproducibility.

At the same time, the use of supplementary material raises several important questions and concerns. What is the appropriate balance between the main text and supplementary information? How is the scientific validity and relevance of supplementary material evaluated during the review process? What is the best method to link supplementary information to the primary paper?

## Why is supplementary material needed? Why is it a problem?

The use of supplementary material is generally more extensive in journals that impose page limits. Compare, for example, papers published in *Bioinformatics* (a journal that strictly controls manuscript length) and *BMC Bioinformatics* (a journal without page limits). At the same time, the extensive use of supplementary material is by no means uncommon even in journals that do not impose manuscript length restrictions. One valid 'excuse' is the need for conciseness in the main manuscript; however, if the effort to squeeze the main findings into a limited space is associated with a lack of attention to the supplementary information, the result may ultimately reduce the clarity of the entire presentation. Paradoxically, despite or maybe because of the large amount of information often available in a supplement, finding and extracting specific points from a supplement can be very difficult – particularly when the supplementary material is effectively a grab-bag of all the analyses that did not make it into the main paper.

Even a cursory examination of papers published in top-tier journals reveals the extent to which supplementary material is used in our field (a summary for the top 10 most highly cited papers in 7 scientific journals is provided in Additional File 1). One can easily find extremes such as these two articles published in *Science*: the first, a 2010 by Werren *et al*. [1], is a 6-page article accompanied by 165 pages of supplementary material. The second, a 2012 paper by Meyer *et al*. [2], is a 5-page article with 144 pages of supplementary material plus a spreadsheet with six additional supplementary tables. In [1], almost half (71 pages) of the supplementary materials contain text supporting (or extending) the information provided in the main manuscript. In addition to the main text, the supplementary material included 210 citations, or 5 times as many as the citations in the main manuscript. In [2], the supplement is organized as 20 separate “Notes”, each with a separate author list and separate first authors and corresponding authors from the main paper. 168 citations are included while the main paper has just 28. These observations are troubling for several reasons; one is that citations within supplementary material do not get tracked by citation indices [3]. The supplementary references generally cite methods that were critical to the study being published. As a result, an important body of work does not receive appropriate recognition – a troubling observation given the increasing use of quantitative impact measures (citation counts, impact factors, etc.) in promotion and funding decisions. Furthermore, science advances through the incremental addition of knowledge to an existing body of work, and the proper acknowledgment of the previous work is a fundamental feature of scientific practice. We are not the first to make this observation (see [3–5]) yet, to our knowledge, neither publishers nor the scientific community have taken any steps towards remedying the situation. If citations within supplementary material are to be allowed, they should be appropriately tracked by citation indices – an impossible proposition today given that most journals do not provide properly formatted online citations for supporting information.

In fact, the majority of journals provide little or no guidance regarding the use of references within supplementary material, in many cases because the initial intent for such material was to enable the addition of data (such as more extensive tables, figures, movies, etc.) rather than supporting text. *Science* was the only journal, among several that we examined, that clearly discussed the issue of references in supplementary material at the time when we originally wrote this article. *Nature* has since also clarified their policy. *Science* requires all references within supplementary material to be included in the main reference list: “*References only cited in the supplementary material should be include at the end of the reference section of the main text, and the reference numbering should continue as if the Supplementary Materials was a continuation of the main text”* [6]. This policy, though apparently useful, is not followed as exemplified by the articles discussed above [1, 2]. *Nature* currently explicitly discourages the use of references in supplementary material: “*Please note that we do not encourage deposition of references within SI as they will not be live links and will not contribute towards citation measures for the papers concerned. Authors who nevertheless wish to post reference lists should continue the numbering from the last reference listed in the print version, rather than repeating the numbering in the print version”* [7]. In fact, both *Science* and *Nature* strictly limit the number of references that can appear in print, a policy that runs directly counter to the very essence of scholarship. Given that references can be provided online for essentially no cost, these policies need to be changed.

## Is supplementary material being reviewed?

Most journals ask reviewers to evaluate supplementary material, either to assess whether the information is necessary, or to actually review it for scientific accuracy. For example, at the journal *Science*, the instructions to authors clearly state: “*To be accepted for posting, supplementary materials must be essential to the scientific integrity and excellence of the paper. The material is subject to the same editorial standards and peer-review procedures as the print publication”* [6]. At the same time, many other journals do not provide any guidance to reviewers, thereby encouraging *ad hoc* reviewing practices that ultimately depend on each reviewer's own decisions.

Despite the instructions provided to reviewers by some journals, supplementary material are rarely reviewed, especially when the length of the supplementary text far exceeds that of the article being published. This fact is well evidenced by the manuscripts highlighted above [1, 2], which are merely two examples among thousands of manuscripts submitted each year with lengthy supplements. Despite the fact that the instructions to authors for the journal *Science* require that all items in the supplementary material be appropriately referenced from the main text, in Werren et al. [1] only 9 out of the 25 supplementary figures, and 17 of the 58 supplementary tables are explicitly mentioned in the main article. The entirety of the 71 pages of supplementary text are referenced through a single citation from the main text (citation 6 in the article), making it difficult for an interested reader or reviewer to even find the specific section being referenced. The fact that this article ignores the journal's own policy strongly suggests that neither the reviewers nor the editors carefully evaluated the supplementary material. We do not wish to single out this article; in fact, we would argue that this example is typical of most high-profile papers published today.

This is a troubling observation as it suggests the possibility that fundamental errors in methods or analyses buried in supplementary files may go undetected, thereby bringing into question the scientific accuracy and validity of the published articles.

## Is supplementary material easy to use?

The primary intent of supplementary material is to provide additional useful information that supports and complements the main text. In addition to figures, the most common form of supplementary information are tables detailing data presented in the main text. These tables are often extensive, containing, for example, information about a large set of genes in an organism. Such information is most useful to readers in a computer readable format (such as tab- or comma-separated plain text files, or a common spreadsheet format). In many cases, though, supplementary tables are provided only in PDF format, thereby significantly hampering the use of these data by researchers attempting to reproduce published results.

Furthermore, as we already highlighted above, the main text is often not well integrated with the supporting information provided in supplementary material. Readers often have to sift through tens or hundreds of pages of text to find information simply referenced from the main text as 'see Supplementary material'.

## A way forward

The situation outlined above is simply unacceptable in today's technologically-advanced world. The limits imposed on the length of articles and their corresponding references derive almost entirely from the constraints of paper-based publication. While these made sense for most of the 20th century, they make no sense at all today, and they distort and even imperil the scientific process. In the 21st century, fewer and fewer scientists peruse paper copies of journals. While one might argue that supplementary material can help improve the presentation of articles, especially in electronic form, the excessive and largely unregulated use of supplementary material is harmful to science. As we discussed above, the scientific quality and validity of supplementary files is rarely evaluated during the review process. Furthermore, cross-referencing prior works is a vital component of the scientific endeavor, yet many scientists' contributions go unrecognized, buried deeply in supplementary files and not tracked by citation indices. This situation disproportionately affects scientists developing the analytical methods that have, in many respects, made the current scientific revolution possible. Authors, reviewers, and journals alike must ensure the adequate acknowledgment, within every scientific article, of all prior work relevant to the study being published.

The ubiquitous use of electronic media in modern scientific publishing provides an opportunity for the better integration of supplementary material with the primary article. Specifically, we propose that supplementary items, irrespective of format, be directly hyper-linked from the text itself. Such references should be to specific sections of the supplementary material rather than the full supplementary text. Mechanisms for providing such links are available in virtually all commonly used word processors, as well as in the commonly used display media (HTML, PDF, etc.), thereby requiring no additional infrastructure to be put into place. The availability of the supplementary information just 'one click away' would not only dramatically improve the utility of published scientific articles, but also increase the likelihood that supplementary material are adequately evaluated during the review process.

Some journals have already taken steps towards providing a rich interface to their articles, and in many cases the supplementary tables, figures, or other media are appropriately hyperlinked directly from the manuscript. In PNAS, for example, online articles are presented in a feature-rich format that includes several useful interactive items: (i) hovering on a citation retrieves the citation in a pop-up widget; (ii) figures and table references are hyperlinked to the actual display item; (iii) files containing supplementary tables and other data are directly hyperlinked from the manuscript, allowing readers to download these items with a single click. In PNAS, these features are also preserved in the PDF version of the articles, and furthermore the supplementary material is automatically included within the downloaded PDF. In most other journals supplementary material must be downloaded separately.

In addition, we believe that removing arbitrary article size limits, at least for the online versions of articles, would have an important impact on removing the artificial distinction between supplementary material and the main manuscript text. An interesting compromise in this direction is exemplified by *Nature Methods*, where articles are accompanied by an Online Methods section that appears in both the online version of the article and the downloaded PDF.

In our discussion above we have singled out two manuscripts published in *Science*, primarily because *Science* is one of the few journals that provides clear instructions to authors and reviewers on supplementary material, yet articles published in this journal frequently overuse supplements. A more extensive analysis of supplementary materials across journals is beyond the scope of this editorial, however interested readers can examine such an analysis recently done for environmental science journals [5], as well as our own survey of 70 highly cited genomics papers from 7 different journals (Additional file 1: Table S1).

Given the extensive use of supplementary material, and the potential harm it poses to science, it is critical that all scientific journals develop clear and consistent policies on the use and review of supplementary material. Some initial recommendations on the use of supplementary material were recently outlined in a report of the National Information Standards Organization and the National Federation of Advanced Information Services [8], but these recommendations still need to be implemented and refined to ensure the ethical and consistent use of supplementary material in our discipline. We hope our paper will motivate scientists and publishers to enact desperately needed changes in the way supplementary materials are evaluated and used in scientific publishing.

## Additional file

Additional file 1: **Spreadsheet containing summary statistics about supplementary material use in 7 scientific journals.** Data is presented for 10 genomics papers from each journal selected based on their number of citations during 2010–2011.
